# Nonleaching Antibacterial Concept Demonstrated by In Situ Construction of 2D Nanoflakes on Magnesium

**DOI:** 10.1002/advs.201902089

**Published:** 2019-09-30

**Authors:** Guomin Wang, Wenjuan Jiang, Shi Mo, Lingxia Xie, Qing Liao, Liangsheng Hu, Qingdong Ruan, Kaiwei Tang, Babak Mehrjou, Mengting Liu, Liping Tong, Huaiyu Wang, Jie Zhuang, Guosong Wu, Paul K. Chu

**Affiliations:** ^1^ Department of Physics Department of Materials Science and Engineering and Department of Biomedical Engineering City University of Hong Kong Tat Chee Avenue Kowloon Hong Kong China; ^2^ College of Pharmacy Western University of Health Sciences 309 E. Second St Pomona CA 91766 USA; ^3^ Research Center for Biomedical Materials and Interfaces Shenzhen Institutes of Advanced Technology Chinese Academy of Sciences Shenzhen 518055 P. R. China; ^4^ Department of Chemistry and Key Laboratory for Preparation and Application of Ordered Structural Materials of Guangdong Province Shantou University Guangdong 515063 P. R. China; ^5^ USC Stevens Neuroimaging and Informatics Institute Keck School of Medicine of USC University of Southern California Los Angeles CA 90033 USA; ^6^ Suzhou Institute of Biomedical Engineering and Technology Chinese Academy of Sciences Suzhou 215163 P. R. China; ^7^ College of Mechanics and Materials Hohai University Nanjing 211100 P. R. China

**Keywords:** antibacterial, magnesium, nanoflake, nonleaching

## Abstract

In bone implants, antibacterial biomaterials with nonleaching surfaces are superior to ones based on abrupt release because systemic side effects arising from the latter can be avoided. In this work, a nonleaching antibacterial concept is demonstrated by fabricating 2D nanoflakes in situ on magnesium (Mg). Different from the conventional antibacterial mechanisms that depend on Mg^2+^ release and pH increase, the nanoflakes exert mechanical tension onto the bacteria membranes to destroy microorganisms on contact and produce intracellular stress via physical interactions, which is also revealed by computational simulations. Moreover, the nanoflake layer decelerates the corrosion process resulting in mitigated Mg^2+^ release, weaker alkalinity in the vicinity, and less hydrogen evolution, in turn inducing less inflammatory reactions and ensuring the biocompatibility as confirmed by the in vivo study. In this way, bacteria are killed by a mechanical process causing very little side effects. This work provides information and insights pertaining to the design of multifunctional biomaterials.

## Graphical abstract

**Nanoflakes are fabricated that stretch bacteria on contact** with assistance of the zigzag structure on the edge. The mechanical stress imposed on bacteria triggers a series of reactions such as membrane rupture and reactive oxygen species burst leading to bacterial death. Biomaterials with the nonleaching antibacterial ability are very promising in implants and cause little side effects and inflammation in vivo.

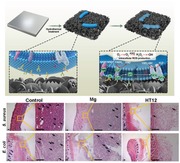

Colonization of microorganisms and subsequent formation of biofilms on metallic implants severely compromise biological functions, lead to surgical failure, and cause excessive patient trauma.^1^ Hence, biomedical implants with intrinsic antibacterial surfaces are important as bacteria prefer to inhabit a solid surface than a planktonic environment.^2^ Among the various surface functionalization strategies, antibacterial surfaces are primarily categorized as leaching surfaces with systemic effects^3^ and nonleaching surfaces that kill bacteria locally.^4^ The former is normally less desirable due to issues such as the potential toxicity of the leached agents and needs to replenish the antibacterial agents if long‐term effects are necessary.^5^ In this respect, bacteria killing on contact without the use of chemicals is simpler and produces less detrimental side effects.^6^

The ability of nanostructures to kill bacteria by physical means and subsequent antibacterial effects have aroused much interest.^7^ For instance, the sharp edges of graphene‐based materials can induce membrane stress in bacteria and single‐wall carbon nanotubes can damage the morphology of *Escherichia coli* mechanically.[qv: 2b,7,8] Some nature‐inspired surfaces have been demonstrated to possess intrinsic mechano‐bactericidal activity.^9^ However, up to now, only scattered success has been reported and the underlying mechanisms are not well understood. In addition, it is challenging to design biomaterials possessing not only inherent bacteria resistance, but also multiple functionalities such as corrosion resistance, biocompatibility, and nontoxicity.

Magnesium (Mg) alloys with excellent mechanical/physicochemical properties and natural biodegradability have been explored in orthopedics.^10^ Mg alloys also have the inherent antibacterial ability arising from leaching of Mg ions due to natural degradation leading to elevation of local pH and hydrogen production.^11^ However, it is difficult to control the degradation rate and so clinical adoption of Mg‐based implants has been hampered.^12^ Surface modification of Mg has been demonstrated to retard the corrosion process but poor adhesion of the foreign layer and introduction of extraneous elements can cause problems related to biosafety and may even undermine the antibacterial ability.^13^ Recently, hydrothermal treatments have been proposed and a 2D morphology can bolster the anticorrosion ability of Mg alloys.^14^ As Mg(OH)_2_ 2D‐nanoflakes are aligned similarly as the aforementioned graphene‐based nanomaterials and bacteria are susceptible to mechanical forces,[qv: 2b,7–8,15] we believe that a nonleaching antibacterial mechanism can be implemented on biomaterials while simultaneously providing improved corrosion resistance and biocompatibility. Although textile samples coated with Mg(OH)_2_ nanoplates have shown zones of bacteria lysis,^16^ to the best of our knowledge, neither a systematic investigation about the antibacterial behavior of Mg(OH)_2_ nanoflakes in situ fabricated on bulk Mg nor comprehensive study on the underlying mechanism has been realized. In addition, a better understanding of the inter‐related effects of nonrelease bacteria killing, corrosion resistance, and biocompatibility is important to the development of biomaterials with tunable multifunctionalities.

In this work, the 2D nanoflakes are produced in situ on Mg by a hydrothermal process and the antibacterial activity, corrosion resistance, as well as biocompatibility are confirmed systematically both in vitro and in vivo. The mechanism is analyzed from the physicochemical, electrochemical, and biological perspectives in addition to theoretical simulation. Different from the common antibacterial mechanisms of bulk Mg attributed to Mg^2+^ release and local pH elevation, the Mg(OH)_2_ nanoflakes trap the bacteria on contact, stretch the cell membranes by strong surface tension, and trigger stress response including intracellular reactive oxygen species (ROS) bursts. The nanostructured surface converts a leaching‐based antibacterial mechanism to a nonleaching one while promoting the anticorrosion properties and mammalian cell growth at the same time.

As shown in **Figure** **1**a and Figure S1 (Supporting Information), 2D nanoflakes with an area of less than 1 µm^2^ each are formed vertically on the surface of Mg after the hydrothermal treatment. When the treatment time is 4 and 8 h (HT4 and HT8), some parts of the surface are still bare and a loose film is formed. After the hydrothermal treatment for 12 h (HT12), the Mg surface is covered by a dense nanoflake film about 1 µm thick and the morphology of the nanoflakes is preserved after ultrasonic cleaning. The nanoflake layer has a porous structure and large surface area as shown in the magnified scanning electron microscopy (SEM) image and the atomic force microscopy (AFM) images (Figure 1b; Figure S2, Supporting Information). Nanoflakes with a zigzag structure (lower right inset in Figure 1a) increase the surface roughness (Figure 1c) providing an unwelcomed milieu for bacteria growth.^17^ The transmission electron microscopy (TEM) image reveals that the 2D nanoflakes have a lateral size of several nanometers and the high‐resolution TEM (HR‐TEM) image shows a crystalline structure with a lattice spacing of 0.237 nm corresponding to the (101) plane of Mg(OH)_2_ (Figure 1d). X‐ray diffraction (XRD, Figure S3, Supporting Information) shows that besides peaks associated with Mg, diffraction peaks for Mg(OH)_2_ are observed from HT8 and HT12 but not HT4, suggesting that the hydrothermal treatment for less than 4 h is not sufficient to produce the Mg(OH)_2_ coating.^18^ The elemental concentrations are determined quantitatively and the O concentration increases with hydrothermal time (Figure 1e). X‐ray photoelectron spectroscopy (XPS) reveals the presence of Mg, O, and C on the surface of the samples (Figure S4, Supporting Information). The high‐resolution Mg 1s spectra discloses conversion of Mg–Mg in pure Mg to Mg–OH in the hydrothermal samples indicating gradual formation of Mg(OH)_2_ on the surface (Figure 1f). These results confirm formation of 2D Mg(OH)_2_ nanoflakes on Mg after the hydrothermal treatment and the amount as well as quality of the coating can be improved by increasing the treatment time to 12 h.

**Figure 1:**
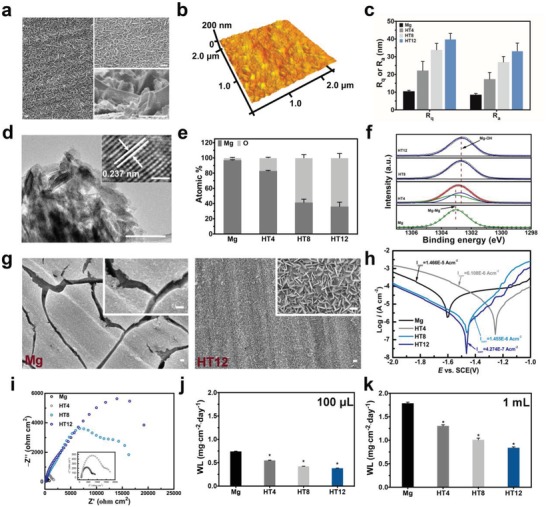
Characterization results and corrosion resistance evaluation: a) SEM images of HT12 with the upper right inset showing the enlarged image and the lower right inset showing the cross‐sectional (45˚) image (Scale bar = 1 µm); b) AFM image of HT12 showing an area of 4 µm^2^; c) Surface roughness values determined by AFM; d) TEM image (Scale bar = 100 nm) of the hydrothermal coating with the inset HR‐TEM image (Scale bar = 2 nm) showing the crystalline structure; e) Elemental contents (Mg and O) determined by EDS; f) High‐resolution Mg 1s XPS spectra; g) Morphology after immersion in the Luria–Bertani (LB) medium for 3 h (Scale bar = 1 µm); h) Polarization curves with the corrosion currents indicated; i) Nyquist plots showing the corrosion behaviour with the lower left inset showing the details of Mg and HT4; Corrosion rates in j) 0.1 mL and k) 1 mL immersion quantitatively determined by weight loss (WL) (* denotes *P* < 0.05 compared to the Mg group).

The anticorrosion properties of the samples are evaluated by immersion and electrochemical tests. Large cracks can be found from the bare Mg after immersion, but the surface of HT12 is intact (Figure 1g). Some nanoflakes detach from the surface of HT4 and HT8 (Figure S5, Supporting Information). The nanoflakes on HT12 are still stable during immersion in a weakly acidic environment (pH = 6) suggesting that HT12 can still work in infected tissues with an acidic pH (Figure S6, Supporting Information). According to the polarization curves in Figure 1h, the corrosion current (*I*_corr_) of bulk Mg is 1.466 × 10^−5^ A cm^−2^ and decreases to 6.108 × 10^−6^ A cm^−2^ after the hydrothermal treatment for 4 h indicating slightly improved corrosion resistance. Corrosion is further mitigated by extending the hydrothermal process to 8 and 12 h and the corresponding *I*_corr_ is lowered by about 90% and 97% (to 1.455 × 10^−6^ and 4.274 × 10^−7^ A cm^−2^), respectively. Electrochemical impedance spectroscopy (EIS) displays the positive relationship between the hydrothermal time and capacitive loop demonstrating enhanced corrosion resistance rendered by the nanoflakes (Figure 1i).^19^ The corrosion resistance of HT groups is quantitatively determined by measuring the weight loss in the 100 µL and 1 mL systems (Figure 1j,k). The weight loss of HT12 is 50% less than that of bare Mg and the weight loss of the 1 mL system exceeds that of the 100 µL system because more Mg^2+^ is needed to saturate the larger volume. The morphological evolution and weight losses are consistent with the electrochemical results corroborating enhanced corrosion resistance after the hydrothermal treatment.

Clinical acceptance of Mg alloys is hampered by hydrogen emission/accumulation and strong alkalization of the surrounding body fluids during fast degradation, which can damage cells and inhibit bone formation. The HT12 group fares better than the Mg group with regard to osteoblast viability (Figure S7a–c, Supporting Information). The cell cytoskeleton of cells is examined by fluorescent microscopy as shown in Figure S7d in the Supporting Information. The cells on HT12 adhere and spread well onto the surface and exhibit some filopodia, whereas those in the Mg group still maintain a round shape and have a lower density, indicating that HT12 favors growth of osteoblasts.

In addition to the good corrosion resistance and biocompatibility, the intrinsic antibacterial properties are crucial to clinical application because bacterial infection is one of the major causes of implant failure. The antibacterial properties of the samples in 100 µL and 1 mL systems are evaluated quantitatively as shown in **Figure** **2**a–c, respectively. In the bacteria culture, the LB medium is used instead of phosphate buffered saline (PBS) to supply a nutrition‐rich environment to energize bacteria.^20^ At 3 h, all the HT groups of the 100 µL system show more bacteria killing than the Mg group and HT12 is the best performer boasting an antibacterial rate of higher than 99%. When the culturing time is increased to 6 h or longer (up to 18 h), the four groups exhibit similar antibacterial activity and most of the bacteria are killed. The antibacterial effects rendered by Gram‐positive and Gram negative bacteria are about the same, although *E. coli* shows better results than *Staphylococcus aureus* due to the different shape and cell wall components.^21^ The results suggest that the antibacterial properties of the HT samples may not stem from release but physically depend on the Mg(OH)_2_ nanoflakes. The antibacterial process is further investigated in the 1 mL system. As shown in Figure 2c, the bare Mg kills 74% of *S. aureus* and 89% of the *E. coli* in the 1 mL system similar to the 100 µL system. In comparison, the HT samples in the 1 mL system underperform with HT12 killing only 49% of *S. aureus* and 60% of *E. coli*, possibly because of less contact between the bacteria and sample surface. Smaller contact is expected to slow the contact‐dependent bacteria killing process. Dead bacteria tend to detach from the surface^22^ and be removed by efferocytosis of macrophages so that the final antibacterial efficiency hardly decreases. The antibacterial rates against these two strains of bacteria are still up to 40%–60% when the system volume is increased to 2 mL (Figure S8, Supporting Information), revealing that the modified Mg alloy is still biodegradable while retaining the nonleaching and leaching processes. As shown in the SEM images in Figure 2d, *S. aureus* and *E. coli* in the control group maintain a normal shape with intact membrane but outer membranous protuberances (white solid arrows) and rupture (white dashed arrows) are observed from the bacteria on Mg, suggesting extreme turgor stress arising from the elevated pH and ion concentration.^23^ In contrast, the bacteria on HT12 exhibit a totally different morphology with *S. aureus* showing concavities (red solid arrows) on the membrane in contact with the nanoflakes. Besides, shriveled *E. coli* cells are found from HT12 and the broken cellular envelopes are stretched by the nanoflakes (red dashed arrows). Compared to the control group, the cell membrane of *E. coli* in the HT12 group is stretched by about 40% as deduced from the SEM images. Owing to the spherical shape and a size smaller than one micrometer, the mechanical interaction between the nanoflakes and *S. aureus* is inferior to that with *E. coli* (90% to 99% antibacterial rates in the 100 µL system for 1 h). Therefore, the membrane change on *S. aureus* is not so typical as that on *E. coli*. TEM discloses that the intracellular substances are distributed uniformly in the control group and plasmolysis takes place in the Mg group possibly due to osmotic stress (Figure S9, Supporting Information).^24^ However, the caved‐in bacteria membranes of the HT12 group indicate a different bacteria inactivation process arising from the physical force exerted on the bacteria by the nanostructure leading to severe deformation of the membrane and eventual death. The results indicate that the process is “mechano‐bactericidal”^9^ instead of following the common release‐dependent antibacterial mechanism of Mg stemming from ion leaching and alkalinity.^12^

**Figure 2:**
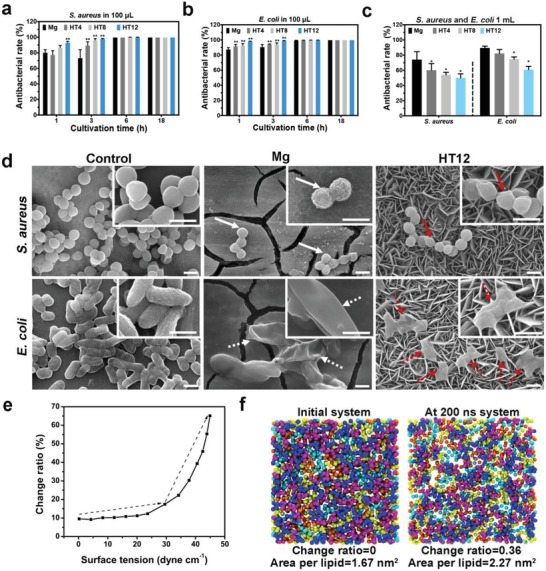
Antibacterial properties test with computational simulation: Time‐dependent antibacterial rates of the different samples in the 100 µL system against a) *S. aureus* and b) *E. coli*, respectively; c) Antibacterial rates of the 1 mL system against *S. aureus* and *E. coli* after cultivation for 3 h; d) Morphology of *S. aureus* and *E. coli* cultivated on different samples for 3 h with the deformed morphology marked by the arrows; e) Final change ratio of the bilayer area under increasing surface tension at *t* = 1 µs; f) Top view of the Coarse Grain Model of the bilayer membrane in the van der Waals (VDW) model with only lipid A shown here using the visual molecular dynamics (VMD) software package—XYA: purple, XYB: blue, LP1: Orange, LP2: cyan, LP3: yellow. Scale bar = 1 µm, * denotes *P* < 0.05 and ** denotes *P* < 0.01 compared to the Mg group.

The physical force which results in the morphological change of bacteria is investigated by theoretical simulation. When surface tensions are exerted on a bilayer, the membrane area increases in the first few nanoseconds and reaches a plateau with small fluctuation afterwards (Figure S10, Supporting Information). Furthermore, a larger surface tension leads to bigger membrane area change (Figure 2e). The change increases gently for low surface tension but the final change shows a sharp rise if the surface tension is higher than 30 dyne cm^−1^, which may result in prompt collapse of the membrane when the external tension exceeds the inherent surface tension of the cell membrane.^25^ Specially, a surface tension of 40.5 dyne cm^−1^ broadens the bilayer by about 40% and the area per lipid increases from 1.67 to 2.27 nm^2^ (Figure 2f) mostly because of enlargement of the intermolecular distance. As verified by SEM, a force of 40.5 dyne cm^−1^ is experienced by the HT12 group and so the morphological change on *E. coli* stems from the nanoflakes which provide strong surface tension to stretch the cell membrane severely and kill the bacteria via a nonrelease and physical‐contact mechanism.

The physiological change in the bacteria provides clues about the antibacterial mechanism and fluorescent staining is employed to assess the viability as well as intracellular oxidative stress of the bacteria. After cultivation for 3 h, the bacteria grow well on the control but many of them in the Mg and HT12 groups show red fluorescence (**Figure** **3**a). Comparing the two latter groups, HT12 kills more bacteria as shown in Figure 3b with 67% of the bacteria on Mg and 76% of the bacteria on HT12 showing red fluorescence. Qualitative and quantitative intracellular oxidative stress analyses with positive ROS signals shown by the HT12 group but not in the control group (Figure 3a,c) indicate that HT12 triggers intracellular ROS bursts. As for the membrane integrity, compared to the bacteria without treatment, the membrane potential of the bacteria on HT12 decreases. In particular, the membrane potential of the HT12 group is higher than that of the Mg group (Figure S11, Supporting Information) suggesting less membrane rupture and leakage of intracellular substances (Figure S12, Supporting Information). The results further indicate that antibacterial effect observed from HT12 originates from membrane stretching that subsequently leads to intracellular ROS elevation and nonviability. In comparison, the bacteria in the Mg group encounter a high pH and osmotic pressure which disturbs the intracellular electrolyte balance producing biological responses and finally results in membrane explosion from the inside.

**Figure 3:**
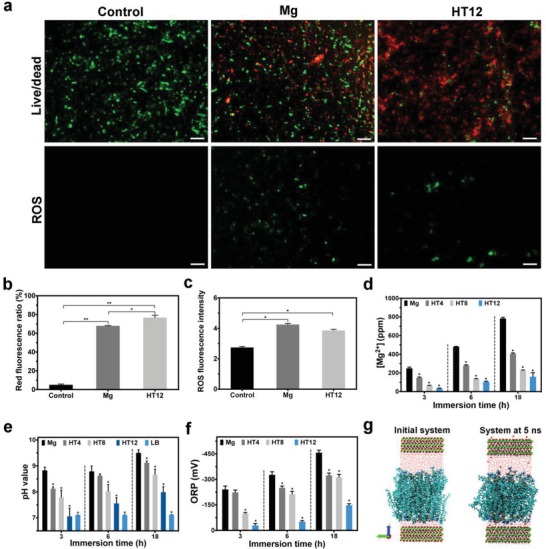
Physiological changes of the bacteria, physicochemical analysis of the LB medium as well as theoretical simulation: a) Fluorescent images showing the viability and intracellular ROS levels of the bacteria on the different samples (Scale bar = 20 µm); Quantitative analysis of b) Bacteria viability and c) ROS intensity by flow cytometry, respectively (* denotes *P* < 0.05 and ** denotes *P* < 0.01 compared to the control group); d) Leaching of Mg^2+^, e) pH, and f) ORP change of the LB medium after immersion for 3, 6, and 18 h (* denotes *P* < 0.05 compared to the Mg group); g) All‐atom model simulation of the dynamic behavior of interfacial water, lipid bilayer, and Mg(OH)_2_ substrate. The Mg(OH)_2_ substrate is shown along the *Z*‐axis outside the periodic boundary box. Atom Color scheme: P: brown, N: blue, Mg: green, Oxygen: red, Hydrogen: white, in the van der Waals (VDW) model. Lipid bilayer POPC is shown in the licorice mode in the visual molecular dynamics (VMD) software package.

To verify the nonrelease mechanism, the LB medium treated with Mg and HT12 is subjected to physicochemical evaluation. Although the amounts of Mg^2+^ leached from all the samples increase with immersion time, less Mg^2+^ release is observed from the HT samples than bulk Mg and the difference becomes more pronounced for a longer hydrothermal time (Figure 3d). Degradation of Mg produces an alkaline environment in the LB medium, but the pH in the HT12 group remains at less than eight even after immersion for 18 h which should not affect the growth of bacteria (Figure 3e).^11^ An alkaline solution tends to have a negative oxidation–reduction potential (ORP). Our results show that the ORP in the Mg group can be more than 200 mV lower than that of HT12 and the difference is larger for longer immersion time (Figure 3f). The negative ORP in the Mg system produces stress to the bacteria causing abnormal ion exchange between the intracellular and extracellular surroundings, triggering abnormal respiration process, and leading to ROS production.^18^ In conjunction with the negative extracellular oxidative stress and inertia of HT12 in the H_2_O_2_ solution (Figure S13, Supporting Information), most bacteria die from direct interactions with the nanoflakes and little physicochemical reactions take place between the medium and nanoflakes. For corroboration, all‐atom MD simulation is performed to investigate the dynamic behavior of the interfacial water, lipid bilayer, and Mg(OH)_2_ substrate and no significant chemical change can be found from the bilayer (Figure 3g) thereby eliminating the possibility of direct chemical reactions between 2D nanoflakes and the bacteria. The results also show that only 7‐OH groups run out from the Mg(OH)_2_ substrate surface in the simulation producing a concentration increase of –OH by only 3.2 × 10^−7^ m, which can hardly alter the pH in the vicinity. All in all, the experimental and theoretical results are consistent and suggest a nonrelease antibacterial mechanism on HT12.

Since infection usually involves biofilm formation, it is crucial to evaluate the anti‐biofilm properties of implants. As shown in Figure S14a (Supporting Information), both Mg and HT12 inhibit biofilm formation after 48 h. 3D confocal scanning laser microscopy (CSLM, Figure S14b, Supporting Information) indicates that most of the bacteria on Mg and HT12 show red fluorescence implying nonviability. Both CSLM and SEM (Figure S14c, Supporting Information) disclose that the bacteria are dispersed sparsely on Mg and HT12 and the membrane is either shrunk or damaged compared to a dense and adherent biofilm on the silicon control. These results confirm the antibacterial and biofilm inhibition effects by the 2D nanoflakes based on a nonrelease mechanism. Furthermore, with less platelets adhering to HT12 than the control and Mg groups, HT12 possesses excellent fouling resistance minimizing the possibility of weakened antibacterial effects in vivo (Figure S15, Supporting Information).

A desirable implant should possess the proper bacteria resistance, anti‐inflammatory ability, and biocompatibility at the same time. Hence, both the Mg and HT12 samples are assessed using a rat model. CFU quantification shows that both Mg and HT12 can kill more than 90% of *S. aureus* and *E. coli* 14 d after implantation (Figure S16a–d, Supporting Information). It should be noted for the HT12 group, some tissue‐related bacteria can still be detected but in general, few bacteria can survive on the implant surface supporting the contact‐dependent antibacterial mechanism. The anti‐inflammatory efficacy is evaluated by micro‐observation of the surgical site and the histological results acquired from the surrounding tissues as shown in Figure S16e in the Supporting Information. It is obvious that serious inflammation occurs for the control group during the entire assessment process and it is most serious on the 10th day (**Figure** **4**a). Although the antibacterial effect of the Mg group is similar to that of the HT12 group, the former shows a severe inflammation process similar to the control group, which can be attributed to the hydrogen burst production, rapid pH increase, and a large amount of Mg^2+^.^26^ Contrarily, the tissues in the HT12 group only exhibit mild inflammation as verified by the unobvious immunological response similar to that of normal tissues (Figure S17, Supporting Information). Besides, the best hair recovery and least tissue swelling observed from the HT12 group (Figure S18, Supporting Information) are consistent with the micro‐observation disclosing no obvious sign of wound inflammation. As shown in Figure S19 (Supporting Information), little morbidity or changes are observed from the main organs of animals corroborating the outstanding biosafety of the HT12 group. The slower corrosion process rendered by the nanoflake layer should be responsible for the better biocompatibility and anti‐inflammatory effects of HT12 compared to the Mg and control groups.

**Figure 4:**
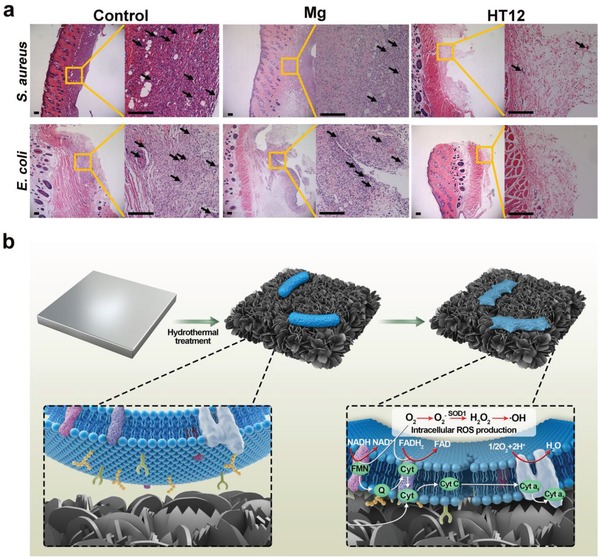
Anti‐inflammation performance and proposed antibacterial mechanism: a) Representative histological analysis of the tissues in contact with the implants by Hematoxylin and Eosin (H&E) staining at 10 d after operation with the enlarged images of the tissues in the yellow square shown on the right side of each image (black arrows indicate significant infiltration of inflammatory cells, Scale bar = 200 µm); b) The proposed antibacterial process based on the results.

Abrupt Mg^2+^ released during natural degradation of Mg‐based implants may produce adverse effects in clinical applications and it is thus important to control the corrosion rate, biocompatibility, and bacterial resistance at the same time. In this work, Mg is treated hydrothermally for different time to produce 2D nanoflakes on the surface to improve all three properties. To the best of our knowledge, this is the first systematic study to employ 2D nanoflakes in situ fabricated on Mg as the antimicrobial platform, which is cost effective and easily scalable to commercial production, and the surface structure has strong adhesion with the substrate boding well for clinical realization. Previous research activities on metal‐based nanoflakes have mostly focused on nanoflakes which are either dispersed or immobilized on the substrate by cross‐linking and deposition,[qv: 8a,27] and so strong adhesion is challenging. In this work, a longer hydrothermal time produces a denser nanoflake layer with more air pockets to block electron transfer^17^ and the Mg(OH)_2_ nanoflakes protect the Mg substrate underneath from water and oxygen thereby retarding natural degradation and minimizing the pH change in the adjacent body fluids. The degradation rates of the hydrothermal Mg diminish in both the neutral and weakly acidic environments and the inflammatory response of the HT12 group is significantly milder than that of the Mg group in vivo. In fact, since a daily intake of 300–400 mg of Mg^2+^ is recommended,^28^ tunable release of Mg^2+^ from Mg‐based implants produced by the hydrothermal treatment can benefit human health in conjunction with wound recovery.^29^ The success of biomedical implants depends largely on initial cell attachment which lays the foundation of ensuing differentiation and matrix production. There are two reasons for the enhanced biocompatibility of HT12. First, the 2D nanoflakes create a suitable environment for tissue cells due to less alkalization surroundings and H_2_ evolution.^30^ Second, the 2D nanoflakes create a desirable topography to mimic the natural environment which impacts adhesion and proliferation of new cells.^31^ Consequently, the materials have outstanding bacterial resistance in conjunction with desirable biocompatibility.^32^

Our results suggest an alternative means to design biomaterials with intrinsic antibacterial properties to lessen the dependence on antibiotics.^33^ The hydrothermal coating on Mg may have been thought to undermine the inherent antibacterial ability of Mg because the coating and air pockets retard Mg^2+^ release. However, the HT samples deliver excellent antibacterial performance as exemplified by the in vitro and in vivo tests. The antibacterial rate of HT12 decreases from 99% in the 100 µL system to 50% in the 1 mL system, but no such difference is found from the Mg group (Figure 2a–c) confirming different antibacterial mechanisms. The different antibacterial mechanism of the HT group in comparison with bulk Mg is investigated from both the biological and physicochemical perspectives in addition to theoretical calculation. The sharp 2D nanoflakes exert a tangential force on the bacteria in contact deforming the bacterial morphology. The structure of the lipid bilayer on the membrane is distorted and intracellular substances are released. Simulation shows that the surface tension that irreversibly deforms the bacteria membrane is about 40 dyne cm^−1^. Membrane deformation also disrupts the respiration chain resulting in abnormal production of intracellular ROS. Different from release of Mg^2+^ and other extraneous antibacterial agents such as silver ions, this antibacterial process arises from mechanical distortion as a result of direct contact between the sharp 2D nanoflakes and bacteria as shown by the physicochemical results (Figure 3d–g). Hence, the HT12 group kill bacteria via a different mechanism which is verified not only experimentally, but also by all‐atom molecular dynamics simulation. The contact‐dependent and nonrelease antibacterial mechanism of HT12 is illustrated in Figure 4b. In this work, we focus on the early implantation period and the Mg alloy is still biodegradable and absorbable after healing. In this way, the nonleaching and leaching processes occur simultaneously. The former is dominant during the early phase at the interface and the latter helps to maintain a weaker antibacterial environment around the implants.

Previous studies have shown that the synergetic effects rendered by Mg^2+^ release and alkalinity lead to bacterial lethality and the mechanism is thus release dependent. Actually, release‐dependent antibacterial processes are the mainstream ones including antibiotics and the bactericidal properties of samples decorated with antibiotics as well as other germicides stem from out‐diffusion of molecules or ions. In spite of the good efficacy in mitigating infection, a large dose or uncontrolled drug release can spur drug resistance and produce adverse side effects. Therefore, the local antibacterial ability based on physical contact, that is, one based on a nonrelease mechanism, is more appealing as many systemic side effects can be circumvented.[qv: 6a] The contact‐dependent and nonleaching antibacterial mechanism demonstrated here is expected to spur the development of multifunctional biomaterials with simultaneous bacterial resistance, corrosion resistance and biocompatibility.

In conclusion, 2D nanoflakes fabricated in situ on Mg is employed as a model to demonstrate a nonleaching antibacterial mechanism which is verified by in vitro and in vivo experiments as well as computational simulation. Different from the conventional antibacterial mechanisms of bulk Mg relying on Mg^2+^ release and local pH elevation, the Mg(OH)_2_ nanoflakes trap the bacteria on contact, stretch the cell membranes by strong surface tension, and trigger stress response including intracellular ROS bursts. The results reveal that it is feasible to produce the suitable surface on Mg to accomplish good corrosion resistance, biocompatibility, as well as nonrelease antibacterial ability at the same time. In addition to the large clinical potential, the concept described here can be extended to the design of other types of biomaterials requiring multiple functions.

## Experimental Section

The materials and methods are available in the Supporting Information. All in vivo tests were approved by the Ethics Committee for Animal Research, Shenzhen Institutes of Advanced Technology, Chinese Academy of Sciences.

## Conflict of Interest

The authors declare no conflict of interest.

## Supporting information

Supplementary
