# Supplementary material for: Nonleaching Antibacterial Concept Demonstrated by In Situ Construction of 2D Nanoflakes on Magnesium
Source: Adv Sci (Weinh). 2019 Sep 30;7(1):1902089. doi: 10.1002/advs.201902089 (PMC6947590; doi:10.1002/advs.201902089)
Supplement: Supplementary file 1 — Supplementary [file ADVS-7-1902089-s001.pdf]

Copyright WILEY-VCH Verlag GmbH & Co. KGaA, 69469 Weinheim, Germany, 2019.

Supporting Information

for *Adv. Sci.,* DOI: 10.1002/advs.201902089

Nonleaching Antibacterial Concept Demonstrated by In Situ Construction of 2D Nanoflakes on Magnesium

Guomin Wang, Wenjuan Jiang, Shi Mo, Lingxia Xie, Qing Liao, Liangsheng Hu, Qingdong Ruan, Kaiwei Tang, Babak Mehrjou, Mengting Liu, Liping Tong, Huaiyu Wang, Jie Zhuang,* Guosong Wu,* and Paul K. Chu**

Copyright WILEY-VCH Verlag GmbH & Co. KGaA, 69469 Weinheim, Germany, 2019.

Supporting Information

Non-Leaching Antibacterial Concept Demonstrated by In Situ Construction of 2D Nanoflakes on Magnesium

*Guomin Wang, Wenjuan Jiang, Shi Mo, Lingxia Xie, Qing Liao, Liangsheng Hu, Qingdong Ruan, Kaiwei Tang, Babak Mehrjou, Mengting Liu, Liping Tong, Huaiyu Wang *, Jie Zhuang *, Guosong Wu *, Paul K. Chu **

Dr. G. M. Wang, S. Mo, Q. D. Ruan, K. W. Tang, B. Mehrjou, Prof. P. K. Chu
Department of Physics, Department of Materials Science and Engineering, and Department of Biomedical Engineering, City University of Hong Kong, Tat Chee Avenue, Kowloon, Hong Kong, China
E-mail: paul.chu@cityu.edu.hk (P.K. Chu)

Dr. W. J. Jiang
College of Pharmacy, Western University of Health Sciences, 309 E. Second St, Pomona, CA 91766, USA

L. X. Xie, Q. Liao, Dr. L. P. Tong, Prof. H. Y. Wang
Research Center for Biomedical Materials and Interfaces, Shenzhen Institutes of Advanced Technology, Chinese Academy of Sciences, Shenzhen, 518055, P.R. China
E-mail: hy.wang1@siat.ac.cn (H.Y. Wang)

Dr. L. S. Hu
Department of Chemistry and Key Laboratory for Preparation and Application of Ordered Structural Materials of Guangdong Province, Shantou University, Guangdong, 515063, P. R. China

Dr. M. T. Liu

USC Stevens Neuroimaging and Informatics Institute, Keck School of Medicine of USC, University of Southern California, Los Angeles, CA 90033, USA

Prof. J. Zhuang
Suzhou Institute of Biomedical Engineering and Technology, Chinese Academy of Sciences, Suzhou, 215163, P.R. China
E-mail: jzhuang@sibet.ac.cn (J. Zhuang)

Prof. G. S. Wu
College of Mechanics and Materials, Hohai University, Nanjing, 211100, P. R. China
E-mail: wuguosong@hhu.edu.cn (G.S. Wu)

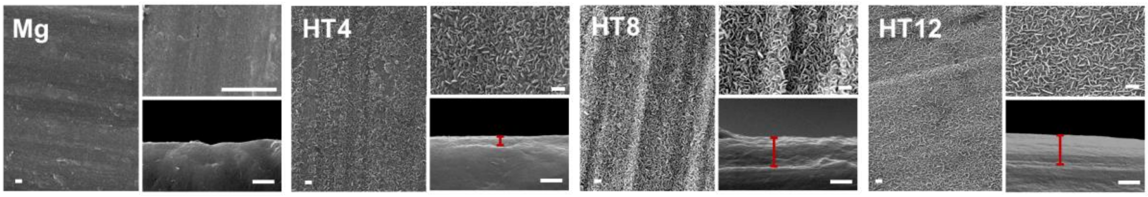

Figure S1. Morphology of the samples determined by SEM (Scale bar = 1 μm).

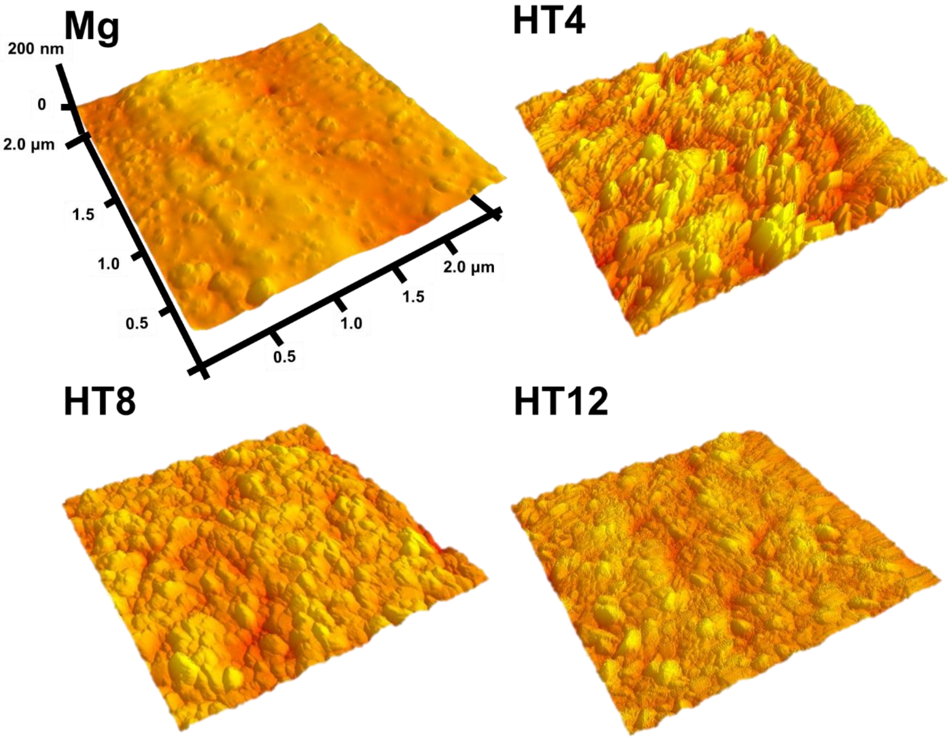

Figure S2. Morphology of the samples determined by AFM.

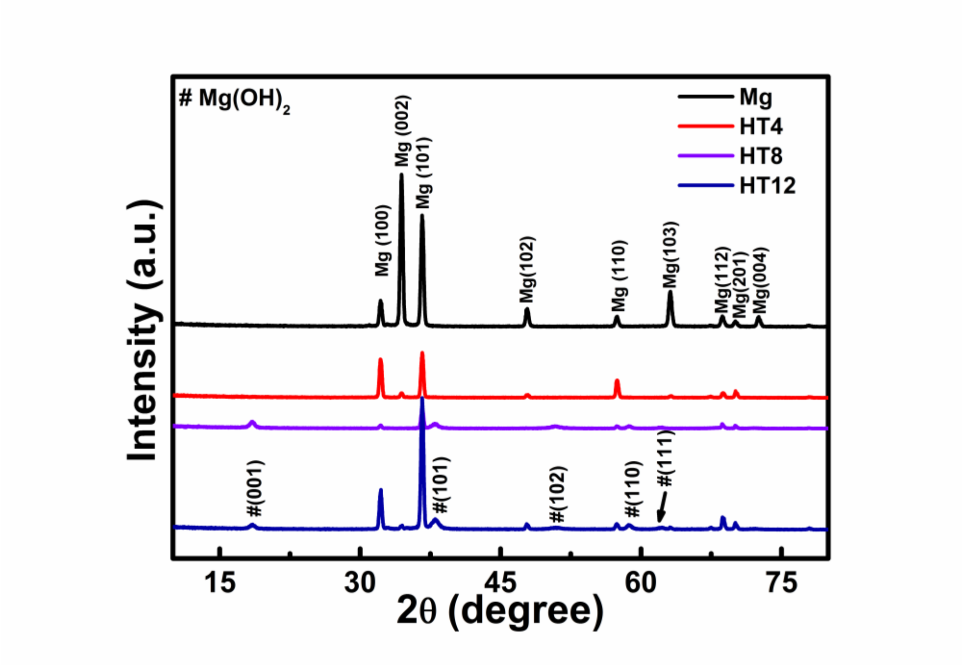

Figure S3. XRD patterns of the Mg and HT samples.

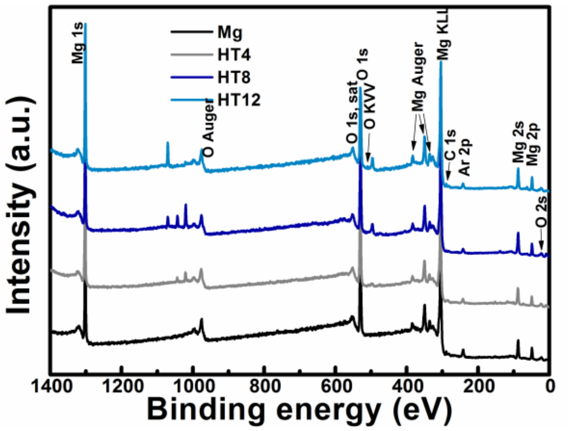

Figure S4. Survey spectra obtained by XPS.

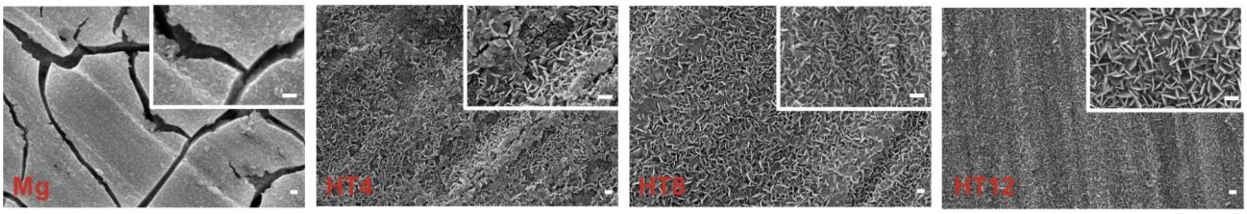

Figure S5. Morphology of the samples after immersion in the LB medium for 3 h (Scale bar = 1 μm).

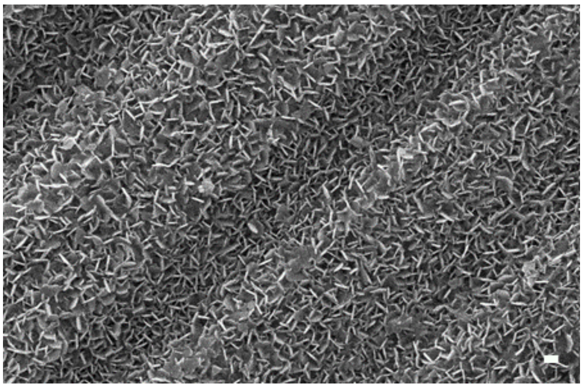

Figure S6. Morphology of HT12 after immersion in the LB medium (pH = 6) for 3 h (Scale bar = 1 μm).

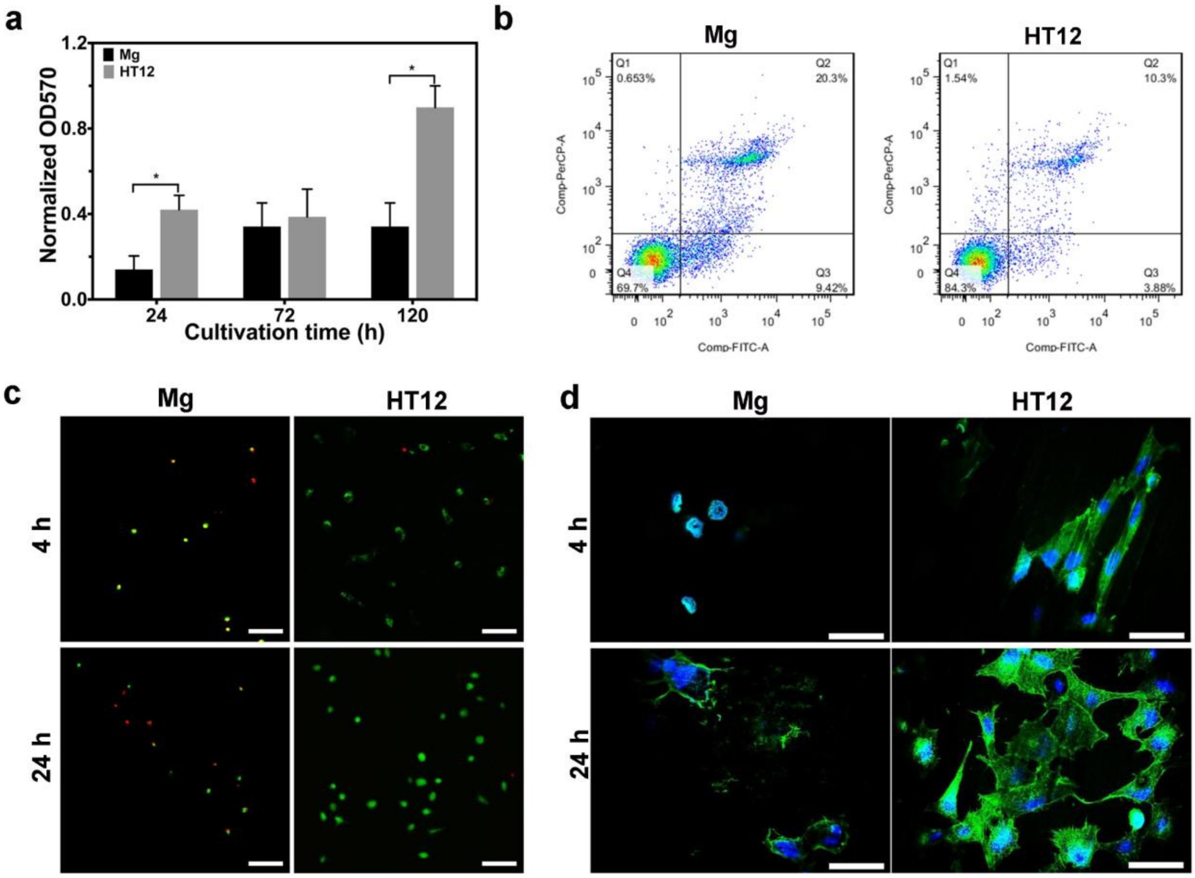

Figure S7. ***In vitro*** **biocompatibility assays:** (a) Quantitative determination of the cell viability at time points of 24, 72, and 120 h with * denoting $P < 0.05$ between Mg and HT12; (b) Flow cytometry (FCM) analysis of osteoblasts cultivated for 24 h with the Q4 quadrants indicating the percentages of normal cells; (c) Real-time viability of osteoblasts on the samples after culturing for 4 and 24 h determined by live/dead staining (Scale bar = 100 μm); (d) Fluorescent images depicting the cytoskeleton of the osteoblasts cultivated on the different samples for 4 and 24 h (Scale bar = 50 μm).

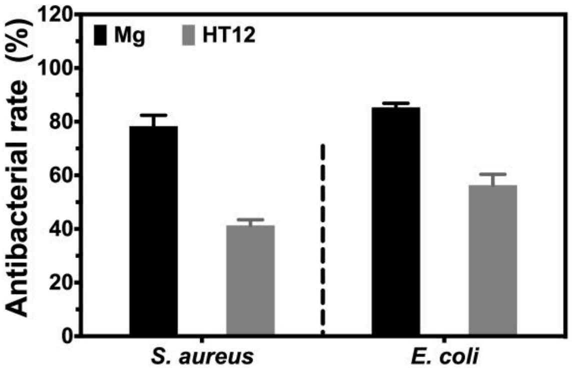

Figure S8. Antibacterial rates of the 2 mL system against *S. aureus* and *E. coli* after cultivation for 3 h.

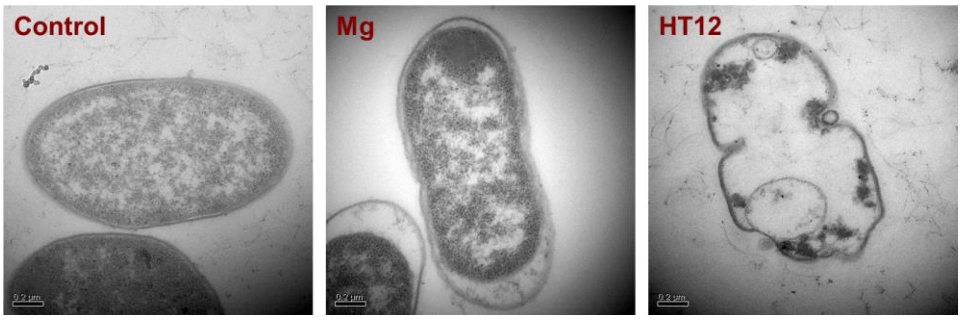

Figure S9. Intracellular and extracellular morphology of *E. coli* cultivated on the samples determined by TEM.

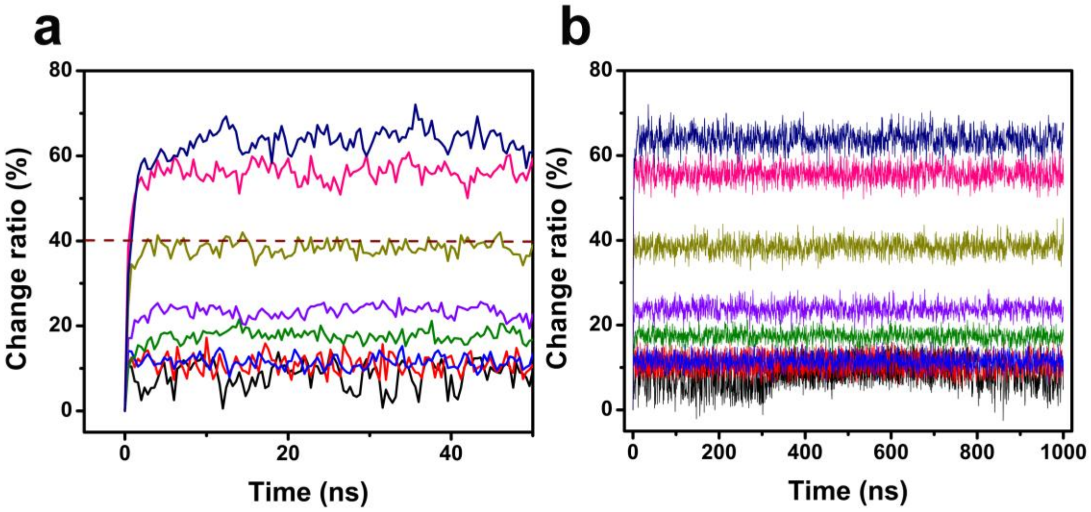

Figure S10. Change ratio of the bilayer area for different surface tensions (black = 0.2, red = 12.2, blue = 20.0, olive = 29.5, violet = 34.2, dark yellow = 40.5, pink = 43.9, and navy = 44.9 dyne cm^{-1}) for simulation time of (a) 50 ns and (b) 1 μs.

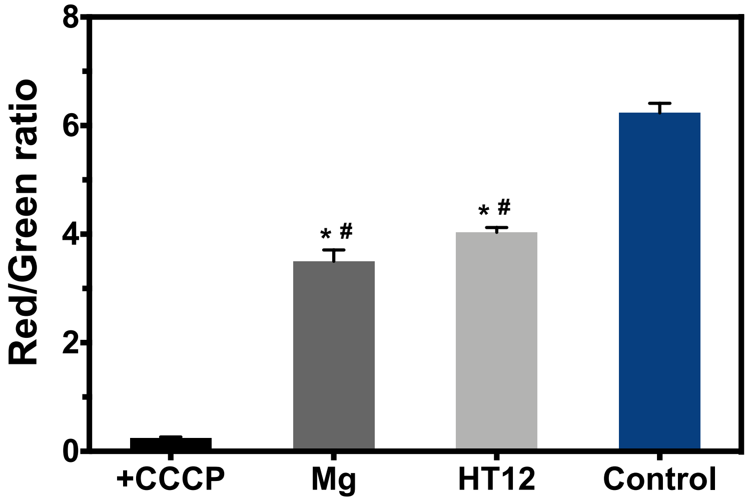

Figure S11. Membrane potentials presented as red/green fluorescence ratios after different treatments (* and # denote $P < 0.05$ compared to the control and +CCCP groups, respectively).

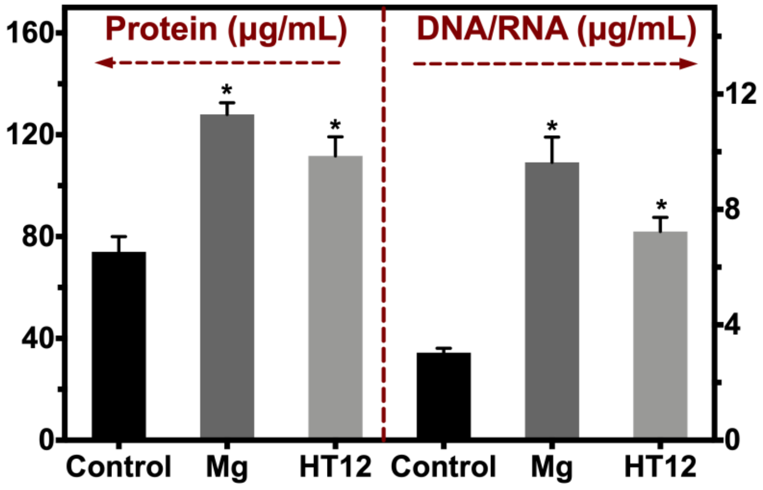

Figure S12. Quantitative comparison of leakage of intracellular substances (protein and DNA/RNA) after different treatments (* denotes $P < 0.05$ compared to the control group).

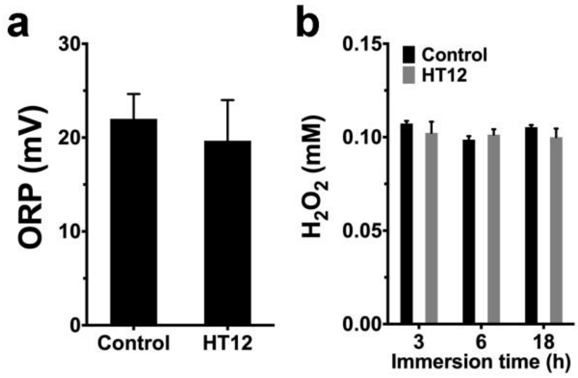

Figure S13. (a) Extracellular oxidative stress evaluation by measuring the ORP of the treated bacteria solution; (b) H_2O_2 concentration during immersion in 0.1 mM H_2O_2.

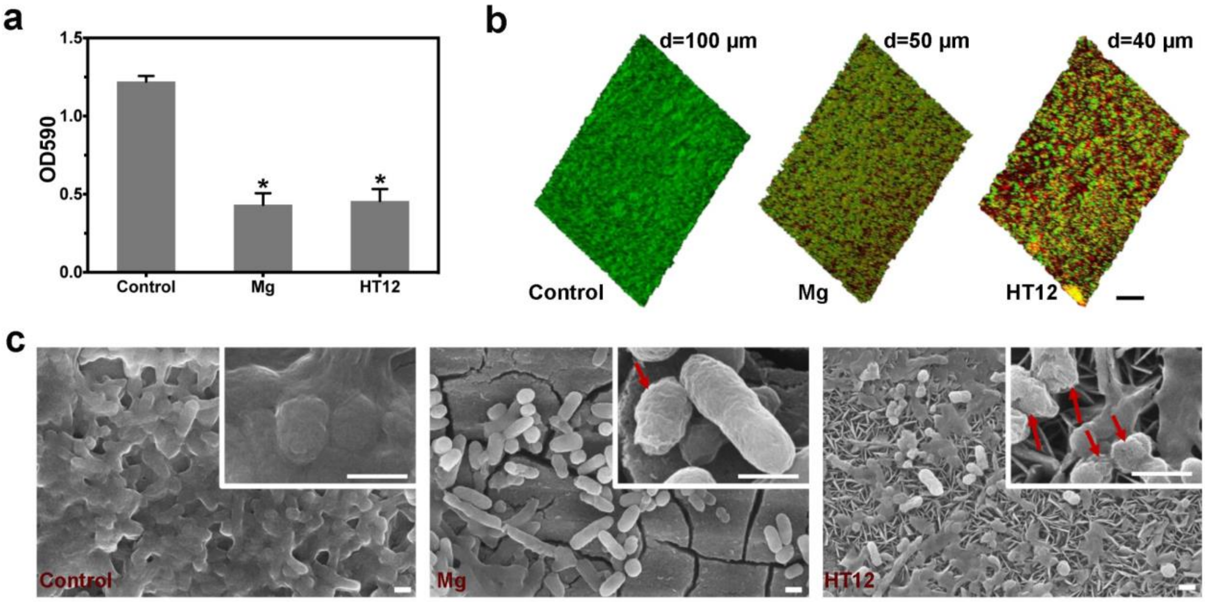

Figure S14. Antibiofilm analysis: (a) Quantitative analysis of the biofilms by crystal violet staining (* denotes $P < 0.05$ compared to the control group); (b) confocal laser scanning microscopy (CLSM) images of the biofilms stained with SYTO 9 (viable bacteria, green fluorescence) and propidium iodide (dead bacteria, red fluorescence) after incubation for 48 h (Scale bar = 100 μm); (c) SEM images of the biofilms showing deformed bacteria marked by red arrows (Scale bar = 1 μm).

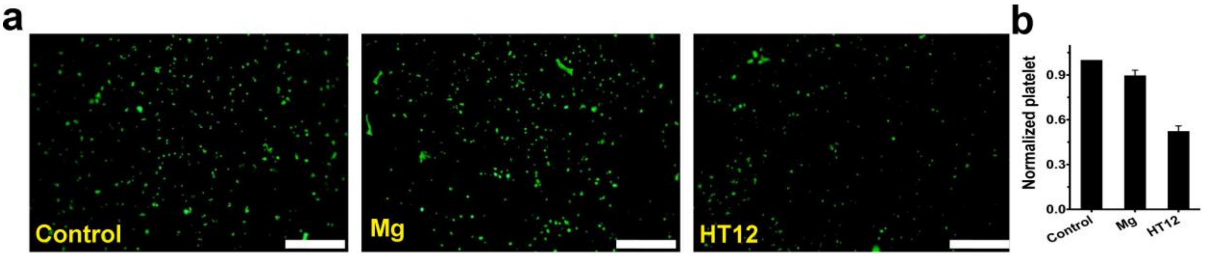

Figure S15. Antifouling performance of HT12: (a) Adhered platelets and (b) Corresponding quantitative results (Scale bar = 100 μm).

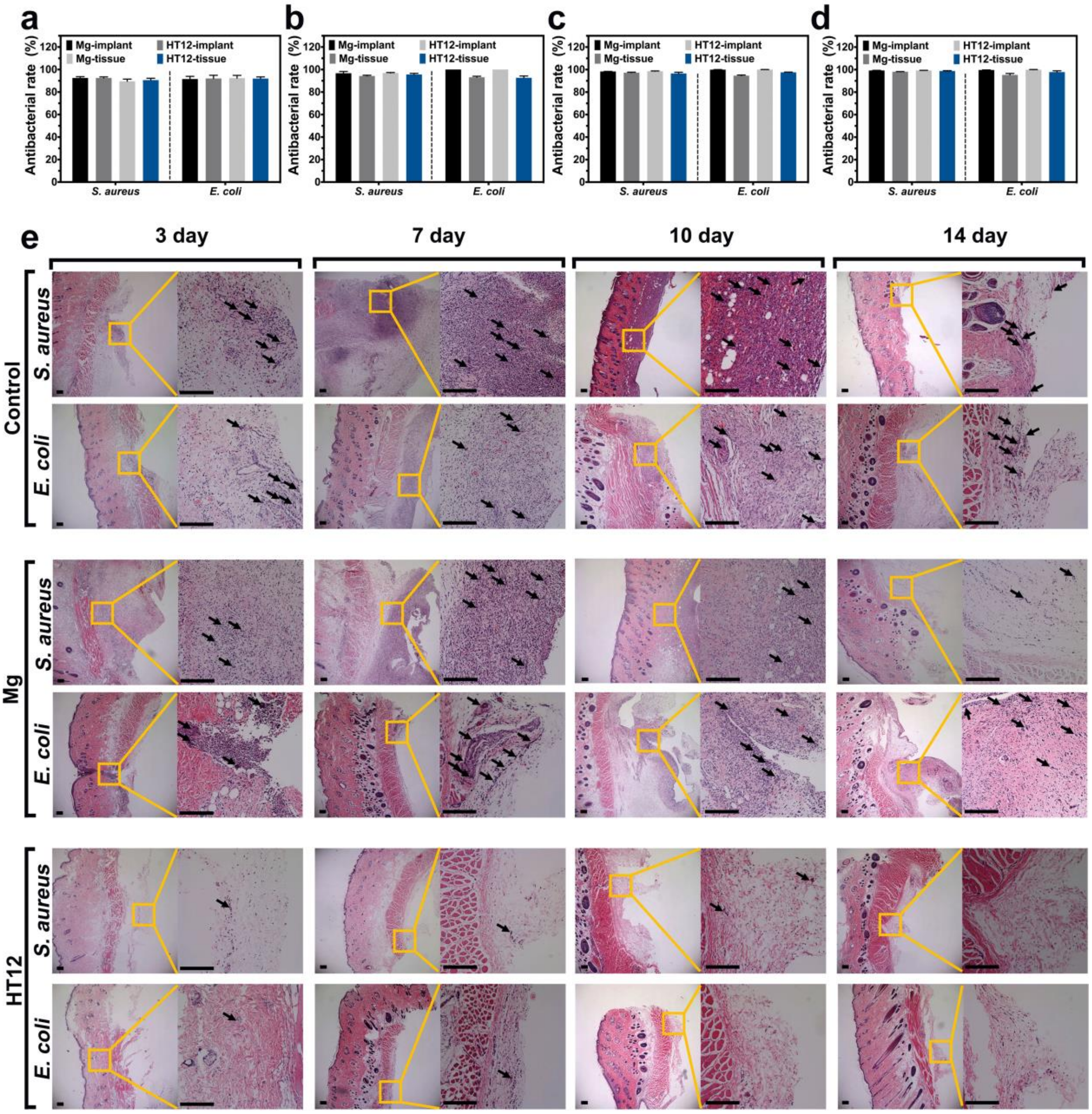

Figure S16. *In vivo* antibacterial and anti-inflammation results: Antibacterial rates of the implant surface and in the surrounding tissues after implantation for (a) 3 d, (b) 7 d, (c) 10 d and (d) 14 d; (e) Histological analysis of the tissues in contact with the implants by Hematoxylin and Eosin (H&E) staining 3, 7, 10 and 14 d after operation with the enlarged images of the tissues in the yellow square shown on the right side of each image (black arrows indicate significant infiltration of inflammatory cells, Scale bar = 200 μm).

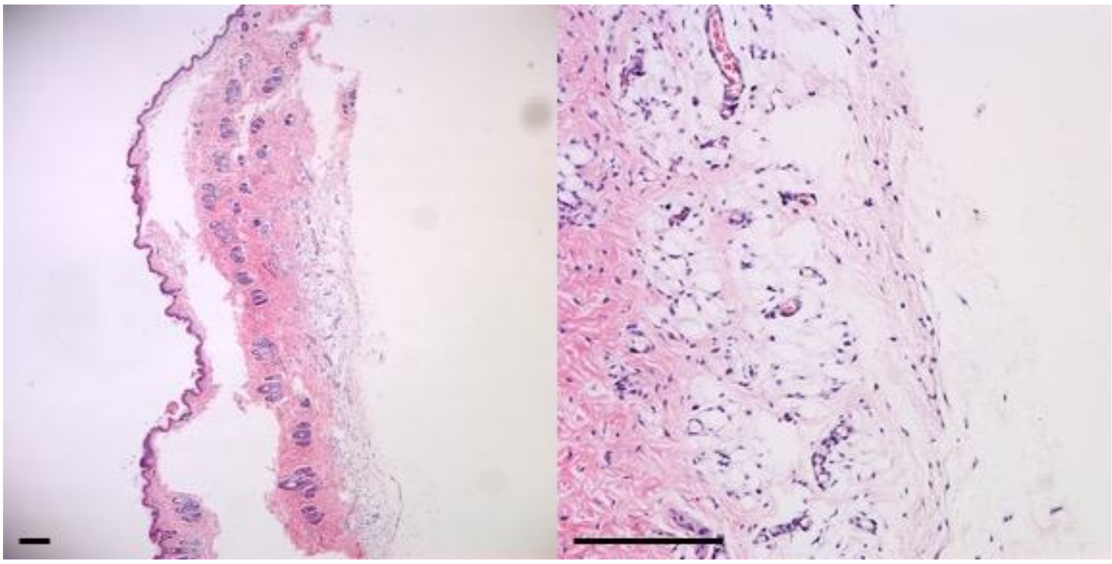

Figure S17. Images of normal tissues (Scale bar = 200 nm).

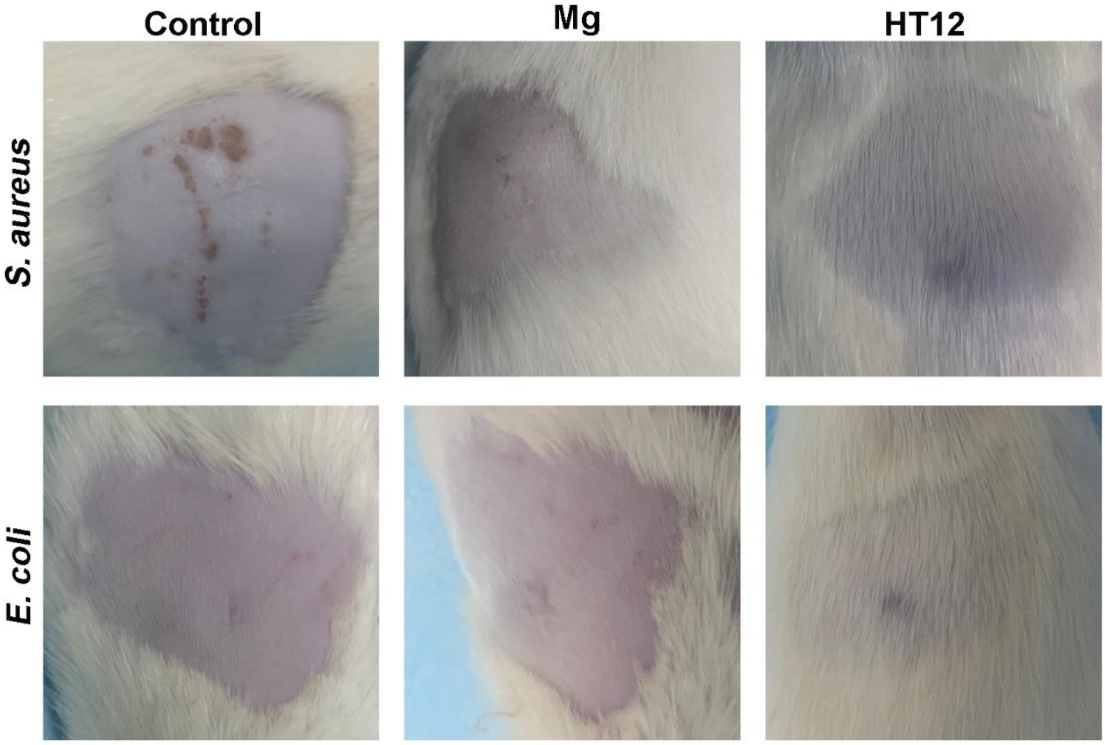

Figure S18. Images of the surgical site 14 days after implantation.

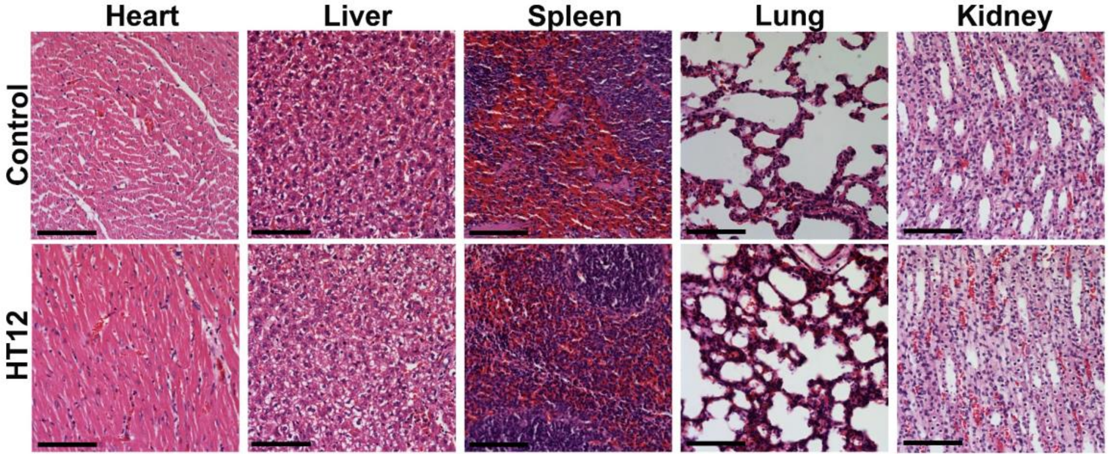

Figure S19. Histological images of the heart, liver, spleen, lung and kidney 14 days after implantation (Scale bar = 100 μm).

Experimental Section

Hydrothermal treatment of Mg. The pure Mg samples (99.8%) were cut into plates with dimensions of 10 mm × 10 mm × 5 mm, ground with SiC abrasive paper (from 400 to 2000 grits) successively, and ultrasonically cleaned in alcohol. After drying in nitrogen, they were put into a 25 mL Teflon-lined autoclave containing 10 mL of NaOH (pH = 12). The autoclave was sealed in a metal shell and treated hydrothermally in an oven at 120 ˚C at a heating rate of 10 ˚C min^{-1} for 4 h, 8 h, and 12 h (samples designated as HT4, HT8, and HT12, respectively). After the reaction, the autoclave was cooled in air and the samples were ultrasonically cleaned with deionized (DI) water, dried in nitrogen, and stored in vacuum until further use.

Materials characterization. SEM (SEM, XL30, ESEM-FEG, Philips, Netherlands), AFM (Veeco MultimodeV, USA), and scanning TEM (STEM, JEM-2010F, JEOL, Japan) were used to observe the morphology and determine the surface roughness of the samples. The structure was determined by STEM and XRD (Siemens D500, Philips, Netherlands) with Cu K_{α} irradiation (λ = 1.54184 Å) at 30 kV and 10 mA. The elemental composition and chemical states were determined by EDS and X-ray photoelectron spectroscopy (XPS, K-Alpha, Thermo Fisher Scientific, USA) with Al K_{α} radiation referenced to the Ar 2p peak at 242.4 eV.

Electrochemical tests. The corrosion tests were carried out in the LB medium at 37 ˚C on an electrochemical workstation (Zennium, Zahner, Germany) with a 3-electrode configuration. The sample with an exposed area of 1 cm^2, platinum rod, and saturated calomel electrode (SCE) were the working electrode, counter electrode, and reference electrode, respectively.

The open circuit potential (OCP) was first monitored as reference prior to the electrochemical test for about 300 s. EIS was conducted at the OCP by applying an alternating potential of 10 mV between 100 kHz and 1 Hz. Afterwards, the samples were dried in nitrogen at room temperature. The Tafel curves were acquired at a scanning rate of 10 mV s^{-1} from -2 V to -1 V with respect to SCE and the degradation rate was determined from the corrosion current (I_{corr}) obtained from the polarization curves by extrapolating the cathodic branch of the polarization curve to the corrosion potential. The data were analyzed and simulated with ZSimpWin and the measurements were carried out at least three times to improve the statistics.

Immersion experiments. The corrosion behavior was evaluated by examining the surfaces and physicochemical properties following immersion. The samples were sealed with silicone rubber with one side (10 mm × 10 mm) exposed and immersed in 1 mL or 0.1 mL of LB on a 24-well plate. The samples were also immersed in the LB medium with a pH of 6 to assess the stability. After 6 h, the samples were taken out, washed with alcohol, dried in nitrogen, and examined by SEM. In the meantime, the leachate solution was collected for the subsequent tests of pH and ORP using a pH meter (STMICRO5, Ohaus, USA) and ORP meter (STORP1, Ohaus, USA). The concentration of Mg^{2+} in the immersion liquid was determined by inductively-coupled plasma mass spectrometry (ICP-MS, Agilent 7500, Agilent, USA) and the sample weight was recorded before corrosion. After immersion for 6 and 24 h, the corrosion products formed on the surface were treated with chromic acid (CrO_3, 200 g L^{-1}; $AgNO_3$, 10 g L^{-1}) for 5 min, rinsed with distilled water and alcohol, and dried in nitrogen. The weight was measured again to calculate the weight loss and degradation rate. The hydrogen peroxide assay kit (ab102500, abcam, UK) was also used to evaluate the behavior of the samples in H_2O_2.

All-atom simulation of $Mg(OH)_2$ (001) with a bilayer. In addition, all-atom molecular dynamics (MD) simulation was performed to investigate the interactions between the hydrothermal coating and solution as described. The trigonal lattice of $Mg(OH)_2$ was adopted as brucite (centrosymmetric space group $P\overline{3}m1$) under ambient conditions. The lattice constants of $Mg(OH)_2$ were $a = b = 3.1486$ Å, $c = 4.7713$ Å, $\alpha = \beta = 90°$, and $\gamma = 120°$ and the supercell had 14 × 14 × 3 crystallographic unit cells corresponding to 588 Mg atoms and 1176 OH groups [1]. The initial coordinates were generated from the in-house MATLAB codes. The fully hydroxylated surface did not have charge redistribution about the OH group on the surface and was aligned parallel to the x-y plane. Above this solid substrate surface was a bilayer 3 Å higher along the z-axis and the bilayer was about 50 Å thick. The simulation box dimensions were 4.25 nm × 4.00 nm × 8.00 nm and the bilayer and solid substrate were placed in an aqueous environment with sodium and chloride ions. The details are listed in Table 1. We chose the modified ClayFF force field [2] to parameterize $Mg(OH)_2$ [3] which had been successfully used for many systems of oxides, hydroxides, and clay minerals [4]. The ClayFF force field considered the entire interactions within and describes the structure completely by the nonbonded Lennard-Jones and Coulomb potentials except for the hydroxyl groups in the clay layer [5].

Regarding the bilayer membrane, the CHARMM36 force field [6] was chosen to model POPC and sodium and chloride ions when interacting with the $Mg(OH)_2$ layers and this force field was also compatible with ClayFF [7]. The simple point charge (SPC) potential was used for water molecules and consistent with both the CHARMM36 and ClayFF force field. The Lorentz-Berthelot mixing rules were applied to both the CHARMM36 and ClayFF force fields for van der Waals interactions in this study.

Molecular dynamics (MD) simulation was performed using GROMACS version 2016.4 [8] with the electrostatic and van der Waals cutoff distance set to 1.2 nm. The periodic boundary conditions were applied to all three dimensions and the similar MD simulation pipeline was adopted through energy minimization using a steepest descents algorithm with convergence obtained once the maximum force on any one atom is less than 100 $kJ\ mol^{-1}nm^{-1}$. Isothermal-isochoric (NVT) and isothermal-isobaric (NPT) equilibration at 310.15 K was performed for 100 ps. NPT used a velocity-rescale Berendsen thermostat, temperature coupling constant of 0.1 ps, and semi-isotropic Parrinello-Rahman barostat with a pressure-coupling constant of 1 ps. Simulation was performed for 5 ns with a 1 *fs* time step. At the same time, the Mg atoms were initially fixed in place by energy minimization, NVT, and NPT steps. The position restraints on the $Mg(OH)_2$ (001) surface were relaxed in the 5 ns n run.

Table 1. Details of the $Mg(OH)_2$ (001) surface with a simple bilayer.

Bilayer	$Mg(OH)_2$		No. of waters	No. of ions		
POPC	Mg	OH	SOL	Na^+	Cl^-	T (K)
48	588	1176	1763	4	4	310.15

***In vitro* antibacterial properties under different conditions.** The pre-sealed samples were sterilized in 75% alcohol for 30 min and dried in nitrogen. In the antibacterial test, Gram-positive (*Staphylococcus aureus*, ATCC29213) and Gram-negative (*Escherichia coli*, ATCC25922) bacteria were chosen as the microorganism models. Both *S. aureus* and *E. coli* were cultured in the LB medium for 12 h in an incubator (shaking rate = 220 rpm and temperature = 37 ˚C) to the exponential growth state. The bacterial solution was doubly diluted with the fresh medium and cultivated for another 3 h for reactivation to achieve OD_{600}

= 0.3 and 0.6, respectively. Afterwards, the bacteria solution was diluted to a concentration of 2-3×10^5 CFU mL^{-1} for the following tests and 100 μL of each solution was added to the surface of the samples. The antibacterial test was carried out on 24-well plates with the gaps between the wells filled with autoclaved water to avoid evaporation of the medium. At time points of 1, 3, 6, and 18 h, the adhered bacteria were detached from the surface with 900 μL of the PBS, diluted to the proper concentration, spread on a solid agar plate, and cultivated for another 16 h to count the CFU. The antibacterial rate was determined by the following formula: Antibacterial rate = $\left(1-\frac{\mathrm{CFU}_{\text{experimental group}}}{\mathrm{CFU}_{\text{control group}}}\right)$ ×100%. The antibacterial effects of the samples in the 1 mL and 2 mL system were determined with the same number of bacteria and more details can be found from our previous paper [9].

In the real-time assessment of the bactericidal effects, the LIVE/DEAD® *Bac*Light™ Bacterial Viability Kit (L-7012, Molecular Probes, Thermo Fisher Scientific, USA) was employed to detect nonviable and viable ones. The dyes were premixed and diluted as stated in the protocol before spreading onto the sample surface and the samples were kept in darkness for 15 min at 37 ˚C. Afterwards, the extra dyes on the samples were rinsed off with PBS. The stained and washed samples were observed under an inverted microscope (Axio Observer.Z1, Zeiss, Germany) with 420-480 nm and 520-580 nm as the excitation and emission wavelengths (green filter) and 480-550 nm and 590-800 nm as the excitation and emission wavelengths (red filter). The dead bacteria fluoresced red while the green ones were deemed viable. The real-time antibacterial rate was determined by calculating the ratio of red fluorescence.

SEM observation of bacteria. SEM was employed to examine the surface morphology of the bacteria cultured on the samples. After incubation for 3 h, the samples with bacteria were

rinsed with PBS three times, fixed with 2.5% glutaraldehyde overnight in the dark, and dehydrated with ethanol with gradient concentrations of 10%, 30%, 50%, 70%, 90%, and 100% (v/v) alcohol sequentially. The specimens were dried in vacuum at 37 ˚C, mounted on the specimen stage, and sputter-deposited with a 10 nm thick Au layer before SEM examination. The bacteria images were taken and the membrane area was calculated.

Coarse Grain (CG) Model for *E. coli* outer membrane. Calculation was performed to determine the ratio of the change of the membrane area for different surface tensions, which mimicked the external surface tension when the membrane was in contact with the 2D nanoflakes. In *E. coli* which is gram-negative, the cell envelop contains a lipopolysaccharide (LPS) rich outer membrane [10] and LPS acting as a selective barrier is crucial to innate immunity in diverse eukaryotic species. Lipid A is a disaccharide-bound lipophilic domain and considered the primary immunostimulatory center of the LPS [11]. In this work, we utilized a coarse grain MARTINI model of *E. coli* outer membrane composed of lipid A in the outer leaflet and 1,2-dipalmitoyl-3-phosphatidyl-ethanolamine (DPPE) in the inner leaflet to investigate the impact of different surface tensions on the *E. coli* outer membrane [12]. Fourteen sets of simulation for the same membrane composition, membrane size, solvent, ions, and temperature but different surface tensions of 0.2, 4.2, 8.3, 12.2, 16.2, 20.0, 23.6, 29.5, 34.2, 37.8, 40.5, 42.5, 43.9 and 44.9 in dyne/cm were performed by the molecular dynamics engine GROMACS version 2016.4 [8] (Table 2). The membrane was built using a versatile membrane-building tool INSANE in python script to construct the CG membrane. The building blocks of *E. coli* lipid A were coded in the freely distributed script and the outer leaflet of the membrane is pure lipid A and the inner leaflet was DPPE. The 10.3 nm × 10.3 nm membrane was solvated with standard water (W) as specified in Table 2 for faster and less expensive simulation. Ten percent of the CG water beads were of the anti-freeze type

and the simulation box dimensions were 10.3 nm × 10.3 nm × 9.69 nm. The system was charge neutral by adding Na^+ counterions and periodic boundary conditions were applied to all three dimensions.

Energy minimization was performed using the steepest-decent algorithm [8a] with a 20 *fs* time until the maximum force on any bead was below the tolerance parameter of 100 $kJ\ mol^{-1} nm^{-1}$. Isothermal-isochoric (NVT) and isothermal-isobaric (NPT) equilibration at 310.15 K was performed for 200 ns. The temperature of each group (membrane and water) was kept constant using the velocity rescale coupling algorithm with a 1 ps time constant. The simulation without surface tension was run for 10 μs with a 20 *fs* time step. The system used semi-isotropic pressure coupling maintained at 1 bar independently in the cross-section of the membrane and perpendicular to the membrane. It was performed by the Parrinello-rahman method with a time constant (τ_p) of 4.0 ps and compressibility of 3.0 × 10^{-5} bar^{-1}. The temperature was maintained at 310 K by independently coupling the lipids and solvent to an external velocity-rescaling thermostat with τ_T = 1.0 ps. The neighbor list was updated every 25 steps using 1.4 and 1.2 nm for short-range van der Waals and electrostatic cutoffs, respectively. The long-range electrostatic interactions were computed using the shifted Coulomb potential with the shift starting at $r = 0$.

After the 10 μs run without surface tension, the system was subjected to 14 different surface tensions by using surface-tension pressure coupling and taking reference pressure as 5 bar, 100 bar, 200 bar, 300 bar, 400 bar, 500 bar, 600 bar, 800 bar, 1,000 bar, 1,200 bar, 1,400 bar, 1,600 bar, 1,800 bar, and 2,000 bar (0.2, 4.2, 8.3, 12.2, 16.2, 20.0, 23.6, 29.5, 34.2, 37.8, 40.5, 42.5, 43.9 and 44.9 in dynes·cm^{-1}, respectively) for coupling in the membrane. These surface tension simulations were performed for 1 μs with a time step of 10 *fs*.

Table 2. Details of the membrane simulation for different surface tensions.

	Inner	Outer	No. of waters	No. of ions	
Species	DPPE	Lipid A	W	Na^+	T (K)
E. coli	192	64	4475	128	310.15

Inner structure of the bacteria examined by TEM. After incubation for 3 h, the specimens were treated ultrasonically for 5 min in PBS to dislodge bacteria from the sample surface. The solution was centrifuged for 5 min (4,000 × g) to collect the bacteria from the bottom. The bacteria were fixed successively with 2.5% glutaraldehyde and 1% OsO_4 at room temperature overnight. After washing with PBS and dehydration with alcohol and acetone with gradient concentrations, the samples were embedded in Spurr's resin (Spurr Embedding Kit, Spurr, USA) before slicing into sections (<100 nm thick) with a glass knife and staining with uranylacetate. The stained samples were placed on a copper wire mesh and examined by TEM (TecnaiG^{2}12 BioTWIN, FEI company, USA) at 120 kV.

Membrane integrity evaluation. The integrity of the bacteria membrane was evaluated by detecting the membrane potential or comparing the leakage of intracellular compounds. The membrane potential of the bacteria was measured by a membrane potential kit (B34950, Invitrogen, USA) and the bacteria treated with CCCP served as the positive control. The bacteria depolarization level was calculated as the red/green fluorescence ratio and more information about the procedures can be found elsewhere [13]. To determine the concentration of the leaked compounds, the samples with bacteria were ultrasonically treated in PBS and centrifuged to acquire the supernatant, and the concentration of the extracellular protein in the suspension was determined by the BCA protein assay kit (Sigma, USA). The concentration of released DNA/RNA was quantitatively measured by detecting the absorbance of the bacteria solution at 260 nm on a NanoDrop spectrophotometer (ND-1000, Thermo Fisher Scientific, USA).

Antibiofilm evaluation. The antibiofilm effects were qualitatively evaluated by CLSM (Leica SPE, Germany) as well as SEM and quantitatively examined by crystal violet staining

[14]. The bacteria with an initial concentration of 2-3 × 10^6 CFU mL^{-1} were cultured on the samples for 48 h. In the quantitative analysis, the specimens were gently rinsed in PBS, stained with 0.1% crystal violet for 20 min, and rinsed in a deionized water bath. The bound crystal violet was eluted with 1 mL of 100% alcohol and then the optical density was determined on a multimode reader (EON, BioTek, USA) at 590 nm [15]. In the qualitative analysis, the samples with adhered bacteria were fixed and dehydrated prior to SEM observation. To determine the bacteria viability and biofilm thickness, the bacteria on the samples were stained by the LIVE/DEAD® *Bac*Light™ Bacterial Viability Kit (L-7012, Molecular Probes, Thermo Fisher Scientific, USA) after cultivation for 48 h. The stained biofilm was observed under a Leica CLSM (Leica TCS SP5 Matrix, Leica Microsystems, Germany) with a 10× oil immersion objective using the aforementioned excitation and emission wavelengths for green and red fluorescence. The images were analyzed by the Leica application suite advanced fluorescence (LASAF) software.

Oxidative stress evaluation. After cultivation for 3 h, the bacteria on the samples were washed with PBS three times, stained with 2', 7'-dichlorodihydrofluorescein diacetate (DCFDA, Beyotime, China) for 15 min in darkness, and rinsed with PBS twice before fluorescent microscopy was performed. FCM (BD FACS Calibur, USA) was employed to quantitatively determine the intracellular ROS levels. The bacteria cultured on the various samples were collected and centrifuged at 4,000× g for 5 min before staining with DCFDA for 15 min in darkness. The stained bacteria were tested in FCM before rinsing with PBS twice and the excitation light wavelength was 488 nm. The X Geo mean data of FL1-H was used to semi-quantitatively compare the fluorescence intensities from the bacteria after different treatments. The ORP test described previously was employed to determine the extracellular oxidative stress of the bacteria solution.

***In vitro* biocompatibility and biosafety assessments.** MC3T3-E1 osteoblasts obtained from the cell bank of the Chinese Academy of Sciences were used to study the biocompatibility of the samples *in vitro*. The cells were nurtured with the culture medium containing dulbecco's modified eagle medium (DMEM) and 10% fetal bovine serum (FBS) and incubated in a humidified atmosphere of 5% CO_2 at 37 °C with the medium refreshed every other day. The cells in the logarithmic growth phase were harvested, centrifuged for 5 min, and diluted to 2×10^4 cells mL^{-1}. The samples were disinfected and 1 mL of the cell solution was seeded on a 24-well plate. The MTT assay was employed to evaluate the cell viability. After cultivation of 24, 72, and 120 h, the medium on the 24-well plate was removed and 1 mL of the MTT solution was added to each well. After further incubation for 4 h, the MTT solution was replaced with the DMSO solution to dissolve the formazan crystals. Subsequently, 100 μL of the solution were transferred to a 96-well plate and the optical density at 570 nm was measured on a multimode reader (EON, BioTek, USA) with DMSO as the negative control. The biocompatibility was visually determined with the aid of the Live/Dead Viability/Cytotoxicity Assay Kit (L-3224, Molecular Probes, Thermo Fisher Scientific, USA). After culturing for 4 and 24 h, the cells were rinsed twice with PBS and 500 μL of the live/dead staining solution were added to each well. After incubation at 37 ˚C with protection from light for 30 min, the samples were observed under a fluorescence microscope (Leica TCS SP5 Matrix, Leica Microsystems, Germany) and more details about the procedures can be found elsewhere [16].

Fluorescent staining was utilized to observe the cytoskeleton of the cells cultivated on the samples for 4 and 24 h. The cells were rinsed with PBS twice, fixed with 4% paraformaldehyde, permeabilized with 0.2% Triton X-100 (Sigma, USA), stained with phalloidin-fluorescein isothiocyanate (Sigma, USA) for 60 min, and stained with 4',6-diamidino-2-phenylindole (DAPI, Sigma, USA) for another 5 min before observation under

the aforementioned inverted fluorescence microscope. The Annexin V-FITC apoptosis detection kit (C1062M, Beyotime, China) was used to determine cell apoptosis after incubation for 24 h. The cells harvested from the various groups were added with 5 μL Annexin V-FITC and 5 μL of propidium iodide (PI) before incubating for 15 min in the absence of light. The apoptosis distribution was determined by FCM at an excitation wavelength of 488 nm.

Biofouling resistance assessment. The whole blood obtained from the rats were centrifugated at 1500 × g for 15 min to collect the platelet-rich plasma (PRP). 150 μL of PRP were added to the surface of each sample and the samples were kept in a standard cell culture incubator for 2 h before washing with PBS three times. Afterwards, the samples were fixed with paraformaldehyde (4%) for 10 min, rinsed with PBS three times, and stained with phalloidin-fluorescein isothiocyanate (Sigma, USA) for 30 min before observation by fluorescent microscopy. The images were captured and fouling resistance effect was quantitatively evaluated by counting the adhered platelets in the specific region.

***In vivo* assessment of the antibacterial performance, anti-inflammatory effects, and biocompatibility.** Sprague Dawley rats (200 g, Female) were employed and the *in vivo* tests were approved by the Ethics Committee for Animal Research, Shenzhen Institutes of Advanced Technology, Chinese Academy of Sciences. The rats were housed for one week to acclimate to the new environment. They were divided into 4 groups assigned as Normal, Control, Mg, and HT12, respectively. They were anesthetized with pentobarbital sodium (45 mg kg^{-1}) *via* intraperitoneal injection before the hair was shaved from a 3 cm × 5 cm area and sterilized with povidone iodine. After incising the skin layer-by-layer parallel to the spine, the samples (5 mm × 5 mm × 2 mm) were implanted in the subcutaneous soft issue. The skin

incisions were sutured before *S. aureus* and *E. coli* in 100 μL of PBS (10^7 CFU mL^{-1}) were injected around the implant. The inflammatory response was examined on a daily basis. After 3, 7, 10, and 14 days, the rats were euthanized. The implants were collected, put in PBS, and shaken for 2 min on a vortex shaker to count the implant-related CFU. Meanwhile, the surrounding soft tissues were immersed in PBS and homogenized (Scientz-IID, Ningbo, Zhejiang, China) for CFU counting. In the histological observation, other parts of the soft issues were fixed with 10% buffered formalin, washed with PBS, dehydrated in gradient alcohol, embedded in the paraffin, and sectioned. The sections were deparaffinized and stained with H&E before observation by optical microscopy. In the toxicity evaluation, the major organs such as heart, liver, kidney, lung, and spleen were harvested at the time of ex-plantation and prepared similar to the historical observation.

Statistical analysis. The data were analyzed by the Student *t*-test and presented as mean ± standard deviation (SD) with a difference of $P < 0.05$ being significant and $P < 0.01$ being highly significant.

References for Supporting Information

[1] V. Y. Kazimirov, M. Smirnov, L. Bourgeois, L. Guerlou-Demourgues, L. Servant, A. Balagurov, I. Natkaniec, N. Khasanova, E. Antipov, *Solid State Ionics* **2010,** 181, 1764.

[2] R. T. Cygan, J.-J. Liang, A. G. Kalinichev, *The Journal of Physical Chemistry B* **2004,** 108, 1255.

[3] a) X. Ou, J. Li, Z. Lin, *The Journal of Physical Chemistry C* **2014,** 118, 29887; b) X. Ou, Z. Zhuang, J. Li, F. Huang, Z. Lin, *RSC Advances* **2016,** 6, 31507.

[4] a) P. S. Braterman, R. T. Cygan, *American Mineralogist* **2006,** 91, 1188; b) T. R. Zeitler, J. A. Greathouse, J. D. Gale, R. T. Cygan, *The Journal of Physical Chemistry C* **2014,** 118, 7946.

[5] a) J. Wang, A. G. Kalinichev, R. J. Kirkpatrick, R. T. Cygan, *The Journal of Physical Chemistry B* **2005,** 109, 15893; b) J. A. Greathouse, J. S. Durkin, J. P. Larentzos, R. T. Cygan, *The Journal of chemical physics* **2009,** 130, 134713; c) R. Eldawud, M. Reitzig, J. Opitz, Y. Rojansakul, W. Jiang, S. Nangia, C. Z. Dinu, *Nanotechnology* **2016,** 27, 085107.

[6] B. R. Brooks, C. L. Brooks III, A. D. Mackerell Jr, L. Nilsson, R. J. Petrella, B. Roux, Y. Won, G. Archontis, C. Bartels, S. Boresch, *Journal of computational chemistry* **2009,** 30, 1545.

[7] a) K. Vanommeslaeghe, E. Hatcher, C. Acharya, S. Kundu, S. Zhong, J. Shim, E. Darian, O. Guvench, P. Lopes, I. Vorobyov, *Journal of computational chemistry* **2010,** 31, 671; b) P. Bjelkmar, P. Larsson, M. A. Cuendet, B. Hess, E. Lindahl, *Journal of Chemical Theory and Computation* **2010,** 6, 459; c) L. B. Wright, T. R. Walsh, *J. Chem. Phys.* **2012,** 137, 8; d) T. Underwood, V. Erastova, P. Cubillas, H. C. Greenwell, *J. Phys. Chem. C* **2015,** 119, 7282.

[8] a) B. Hess, C. Kutzner, D. Van Der Spoel, E. Lindahl, *Journal of chemical theory and computation* **2008,** 4, 435; b) S. Pronk, S. Páll, R. Schulz, P. Larsson, P. Bjelkmar, R. Apostolov, M. R. Shirts, J. C. Smith, P. M. Kasson, D. Van Der Spoel, *Bioinformatics* **2013,** 29, 845.

[9] G. Wang, H. Feng, A. Gao, Q. Hao, W. Jin, X. Peng, W. Li, G. Wu, P. K. Chu, *ACS Appl. Mater. Interfaces* **2016,** 8, 24509.

[10] U. Zähringer, B. Lindner, E. T. Rietschel, *Advances in carbohydrate chemistry and biochemistry* **1994,** 50, 211.

[11] a) D. Dixon, R. Darveau, *Journal of dental research* **2005,** 84, 584; b) B. D. Needham, M. S. Trent, *Nature Reviews Microbiology* **2013,** 11, 467.

[12] a) H. Ma, F. J. Irudayanathan, W. Jiang, S. Nangia, *The Journal of Physical Chemistry B* **2015,** 119, 14668; b) H. Ma, D. D. Cummins, N. B. Edelstein, J. Gomez, A. Khan, M. D. Llewellyn, T. Picudella, S. R. Willsey, S. Nangia, *Journal of chemical theory and computation* **2017,** 13, 811.

[13] D. J. Novo, N. G. Perlmutter, R. H. Hunt, H. M. Shapiro, *Antimicrob. Agents Chemother.* **2000,** 44, 827.

[14] G. Wang, H. Feng, L. Hu, W. Jin, Q. Hao, A. Gao, X. Peng, W. Li, K.-Y. Wong, H. Wang, *Nat. Commun.* **2018,** 9, 2055.
[15] A. K. Epstein, T.-S. Wong, R. A. Belisle, E. M. Boggs, J. Aizenberg, *Proc. Natl. Acad. Sci. U. S. A.* **2012,** 109, 13182.
[16] L. Wei, Z. Chen, L. Shi, R. Long, A. V. Anzalone, L. Zhang, F. Hu, R. Yuste, V. W. Cornish, W. Min, *Nature* **2017,** 544, 465.
